# How did vocal communication come to dominate human language? A view from the womb

**DOI:** 10.1371/journal.pbio.3003141

**Published:** 2025-04-15

**Authors:** Alexis Hervais-Adelman, Simon W. Townsend

**Affiliations:** 1 Department of Basic Neuroscience, University of Geneva, Geneva, Switzerland; 2 Zurich Centre for Linguistics, University of Zurich, Zurich, Switzerland; 3 Institute for the Interdisciplinary Study of Language Evolution, University of Zurich, Zurich, Switzerland; 4 Department of Evolutionary Anthropology, University of Zurich, Zurich, Switzerland

## Abstract

Whether human language evolved via a gestural or a vocal route remains an unresolved and contentious issue. Given the existence of two preconditions—a “language faculty” and the capacity for imitative learning both vocally and manually—there is no compelling evidence for gesture being inherently inferior to vocalization as a mode of linguistic expression; indeed, signed languages are capable of the same expressive range as spoken ones. Here, we revisit this conundrum, championing recent methodological advances in human neuroimaging (specifically, in utero functional magnetic resonance imaging) as a window into the role of the prenatal gestational period in language evolution, a critical, yet currently underexplored environment in which fetuses are exposed to, and become attuned to, spoken language. In this Unsolved Mystery, we outline how, compared to visual sensitivity, the ontogenically earlier development of auditory sensitivity, beginning in utero and persisting for several months *post-partum,* alongside the relative permeability of the uterine environment to sound, but not light, may constitute a small but significant contribution to the current dominance of spoken language.

## Abstract 2

Whether human language evolved via a gestural or a vocal route remains an unresolved and contentious debate. This Unsolved Mystery discusses evidence suggesting that fetal auditory development and learning in the womb are important evolutionary factors in the dominance of vocal communication.

## Introduction

Human expressive language is intrinsically biased toward the spoken modality, yet how speech came to be the primary mode of communication remains unclear. One dominant explanation is that language evolved in the gestural modality with spoken language then piggybacking on this existing cognitive architecture and ultimately becoming the dominant communicative modality [1–3]. Classical arguments in favor of gestural evolution of language suggest that, since great apes exhibit more flexible communication in the visual-gestural than vocal modality, our ancestors would have also been gestural communicators [4,5]. An additional argument suggests that in humans, gestural communication begins earlier in infants [6] and, under the premise that ontogeny recapitulates phylogeny, a similar communicative progression may have occurred over evolutionary time.

Whilst perhaps intuitive, the gestural origins hypothesis has recently received substantial challenges from both developmental and comparative perspectives. Firstly, a systematic quantification of the rates of speech-like vocalizations and gestures over early infancy suggests that at 3 months infants produce 35 times more vocal protophonetic than gestural acts [7], indicating that vocalizations, not gestures, are the primary mode of communication in infancy. Secondly, while great apes do indeed deploy their gestures flexibly, this is also true of their vocalizations, and a growing body of evidence suggests that primate vocal communication arguably displays more similarities with key components of human language than gestures [8,9].

These shortcomings of the gesture-first hypothesis are further compounded by the unresolved issue of the precise catalyst promoting a shift from gestural to vocal communication. Numerous benefits of speech over gesture have been proposed, ranging from facilitating communication in the dark or at a distance, to predator avoidance and the potential for communication while leaving the hands free for tool use or infant carrying. Although it cannot be disputed that vocal communication does have these properties, and that they are likely beneficial, an argument that they can drive the evolution of the dominance of the vocal mode has received little compelling empirical support. The practical benefits to the communicative modality cannot serve as explanations for the selection of speech over gesture. Evolution, as it is currently understood, does not proceed on the basis of “possible but as-yet-unrealized advantages” [10]. Rather, changes arise cumulatively, on the basis of gradual shifts that confer benefits for contemporaneous functions. It is difficult, if not impossible, to establish from the existing (fossil) record or from phylogenetic comparisons that the presumed incremental changes from gesture to vocalization was indeed sufficiently adaptive at intermediate stages to drive a selection benefit in its favor.

Here, we argue that the debate so far has neglected to integrate a class of clues from ontogeny that can cast light on the driving factors that have been instrumental in promoting the dominance of the vocal channel. Specifically, we contend that the nature and constraint of the prenatal period, i.e., gestation, play a powerful role in this story. We compare the development and maturation of the auditory and visual systems in utero and outline the evidence in favor of a potent role for acoustic information in tuning a newborn’s *post-partum* behavior. Evidence from emerging signed languages in deaf communities [11] and communities with a high proportion of congenitally deaf members [12] indicates that the necessary preconditions are in place for the development of vocal or gestural expressive language given the appropriate conditions for cultural transmission after birth [13,14]. In addressing the unsolved mystery of why the vocal mode has come to dominate human communication despite the availability of two viable channels, we contend that the combination of the environment in utero and the development of fetal sensory and cognitive capacities during the third trimester of gestation is such that it can support environmentally driven audiomotor learning to a significantly greater degree than visuo-gestural.

## The different ontogenetic timelines of hearing and vision

**Fig 1:**
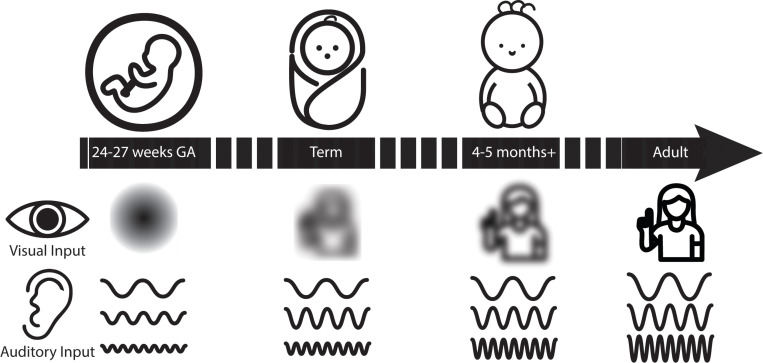
A schematic representation of the timeline of fetal visual and auditory perception. In utero, acoustic stimulation is available, while only luminosity is available to the visual system. At birth, although the physical barrier of the abdomen is no longer in place, the acuity of the fetal visual system is very poor. At several months post-partum, visual acuity has improved but remains poorer than that of adults until approximately 6 months. Meanwhile, the auditory system, despite having relatively elevated thresholds compared to adults, especially in higher frequencies, is nevertheless responsive to a broad range of frequencies (see text for details) from approximately 24 gestational weeks onward.

Our basic premise is that the uterine environment is permissive to auditory signals, and that the human auditory system begins to be responsive to sound at approximately 6 months of gestational age (GA) [15] and increases in its range of frequency sensitivity during gestation [16]. This provides the necessary preconditions for a human fetus to be materially affected by auditory stimuli. In contrast, the womb is poorly penetrant to light, and the human visual system suffers from very poor acuity for the first months of life. The head-start therefore available to the development of auditory-vocal over visuo-gestural communication may constitute an additional significant driving factor in tilting the balance in favor of a vocal mode. Moreover, this relative developmental lag for vision is not confined to the uterine environment—even when the maternal abdomen no longer blocks visual input *post-partum*, the scope for learning about gestures is limited by the acuity of the visual system, which does not reach adult-like levels until between 24 and 36 months *post-partum* (see Fig 1) [17]. All the while, the peripheral auditory system is sensitive and responsive. While it is not feasible to reliably determine neonatal auditory sensitivity from overt behavioral measures, data using auditory brainstem responses suggest that the auditory system is responsive across the frequency range 0.5–8 kHz, albeit with moderately elevated absolute thresholds compared to adults [18]. The vocal channel therefore benefits from a 3-month in utero head-start over the gestural, in addition to a substantial *post-partum* interval during which the visual system provides only poor resolution. We argue that this provides ample basis for a precocious bias of the habitual communication channel in favor of the auditory-vocal over the visuo-gestural.

## The fetal auditory system responds to sound, and extracts and stores regularities

The fetal environment is very different to the adult environment in which acoustic vibrations are transmitted to the ear through air. The fetus develops in a fluid-filled sac inside the mother’s abdomen that shields the fetus from the outside world and also has an attenuating effect on the acoustical information that can reach the fetal ear. Nevertheless, a considerable body of work suggests that all frequencies within the typical human auditory range can be transmitted to the fetus, albeit with an attenuation on the order of 25–30 dB [19]. This implies that all maternal speech and any sufficiently loud speech of others will be transmitted to the fetal cochlea. One study [20] claims that although only approximately 30% of phonetic information from speech in the environment is available to the fetal auditory system, intonation is very well transmitted. The uterine environment features a constant background noise of maternal sounds, particularly the heartbeat and digestive processes, but these are confined to lower frequencies (below 300 Hz) [21] and thus do not mask all other sounds. Recent work using hydrophones affixed to the occiput of fetal sheep in utero has revealed that the transmission of sound from 100 Hz to 20 kHz is moderately attenuated by the uterine environment in the amniotic sac [22], but to a lesser degree with less high-frequency roll-off than previously thought.

**Fig 2:**
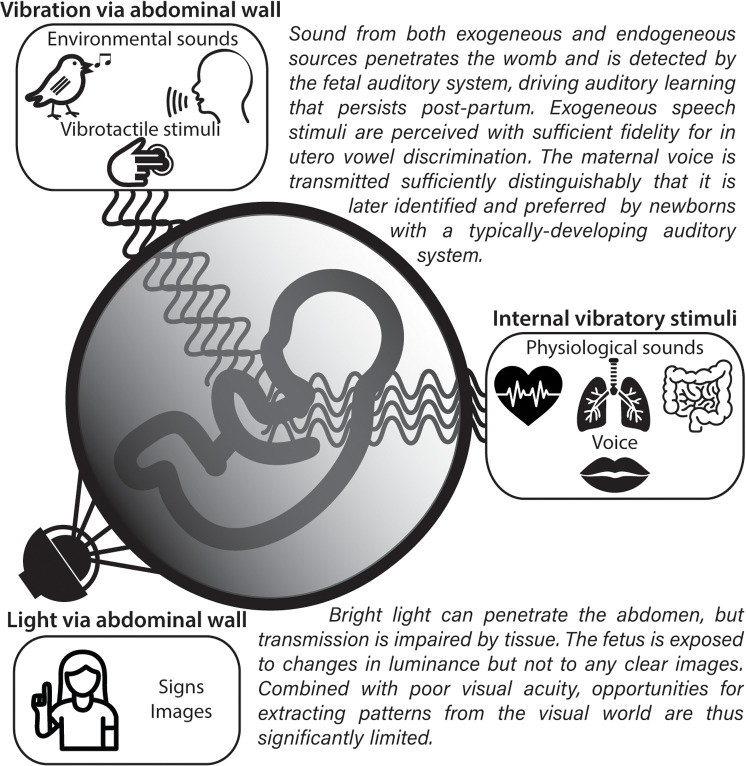
Schematic illustration of sensory input to the fetus. The diagram highlights the relative penetrance of vibrotactile and acoustic stimuli, which can be transmitted directly as pressure waves, compared to light, whose photons are absorbed by the abdomen, reflected, and scattered, preventing the transmission of images. All icons are original works or reused under a CC-BY4.0 license. “Vibrotactile stimuli” icon obtained from https://icon-icons.com/icon/touching-ngers/64385; “heartbeat” icon by M2n obtained from https://thenounproject.com/icon/heartbeat-5770628/.

Empirical evidence indicates that the human fetus has a fully functioning auditory system at 6 months GA [15], suggesting that the fetus should be able to perceive and learn from auditory stimuli in the womb. Indeed, there is overwhelming evidence that fetal auditory learning occurs both in non-linguistic and linguistic auditory domains and is retained *post-partum*. Newborn infants discriminate between their mother’s voice and that of other unknown female speakers [23], between their native language and other languages [24,25], and they even exhibit preferences for melodies [26] or stories [27] to which they were exposed in utero. Further, it has been shown that newborn infants discriminate native and foreign vowels [28], and that they do so in a manner consistent with having begun to develop native vowel categories in utero. Other evidence points to significant in utero learning of the properties of trisyllabic nonwords [29] reflected in *post-partum* neural traces consistent with advanced sequence recognition and pitch discrimination capabilities. It should be noted that testing newborns or infants a few days *post-partum* may carry the risk of some influence of post-natal learning. This is not, however, the case for evidence for auditory sensitivity and habituation that has been acquired in utero. For example, fetal heart rate changes have been recorded in response to airborne auditory stimuli demonstrating patterns consistent with habituation and dishabituation [30]. Even earlier work [31] had already demonstrated fetal heart-rate changes consistent with a discriminative ability at 35 weeks GA for airborne syllable presentation. Further, evidence from in utero magnetoencephalography, a non-invasive technique that measures magnetic field fluctuations induced by neuronal activity, also suggests that the fetal brain produces a classical mismatch response when presented with a novel stimulus in a train of identical stimuli [32,33]. Thus, prior to birth, the fetus processes and derives regularities from the auditory input, laying the foundations for future recognition, categorization and, ultimately, comprehension of speech (see Fig 2). We therefore contend that early experience with speech stimuli, both environmental and maternal in origin has the potential to exert developmental influences on the in utero brain.

## Methodological advances in MRI enable finer-grained insights into the fetal brain

Recent advances in neuroimaging techniques and growing acceptance of the technique have facilitated the take-up of fetal neuroimaging as an investigative (not merely clinical) technique. In utero neuroimaging is a valuable source of information for revealing the structural and functional organization of the fetal brain. Over the past decades, in utero functional imaging has demonstrated that fetal brains respond to acoustic stimulation in auditory cortical areas [34], and display differentiated cerebral responses to the maternal *versus* non-maternal voice [35–37]. Structural and resting-state imaging of fetal brains also provides new insights into the underlying organization of the brain as a whole, and specifically in speech and language-related networks.

The available data on in utero development of auditory and speech circuits has recently been comprehensively reviewed [38] and there is overwhelming evidence that not only does the auditory system respond to stimuli from around six months GA, it is embedded in an increasingly complex fronto-temporal network developing from 26–27 weeks GA onwards [39,40], and that these networks become increasingly adult-like. For example, one study [41] showed a 61.66% overlap between adult and fetal connectomes in the third trimester.

## Prenatal sounds can alter developmental trajectories

Significantly, evidence abounds that prenatal auditory sensitivity provides a potential mechanism for “auditory developmental programming”. It has now been demonstrated that prenatal sounds provoke phenotypic changes in embryos across diverse taxa, including invertebrates, amphibians, reptiles, and birds [comprehensively reviewed in 42], essentially demonstrating that there is a mechanism for tailoring the fitness of an individual to the environment in response to an external acoustic (or vibratory) signal. A number of striking examples of this phenomenon exist. For example, in invertebrates, the synchronized hatching of stinkbugs (*Halyomorpha halys*) in response to an egg-cracking vibration causes all members of a batch to emerge simultaneously [43], with a proposed function of avoiding egg cannibalism by hatchlings. Shield bugs (*Parastrachia japonensis*) are attested to hatch synchronously in response to maternal vibrations [44]. Among amphibians, red-eyed tree frogs (*Agalychnis callidryas*) avoid egg predation by responding to vibrations from approaching predators with immediate early hatching [45], as do multiple species of glass frogs [46]. In birds, multiple examples of *in ovo* learning have been reported, including the acquisition of a vocal “password” by superb fairy wren (*Malurus cyaneus*) chicks [47] from maternal incubation calls. Elements of the calls are reprised by the hatchlings in their begging calls, thereby identifying them as biological brood members, ensuring that they receive food, and reducing the risk of parasitism from cuckoo chicks unfamiliar with the song. There are even more potent experimental demonstrations of the extent to which prenatal sound exposure can alter individual phenotypes directly. In one recent demonstration, prenatal (*in ovo*) exposure to “heat-calls” in arid-adapted zebra finches was shown to drive development of superior heat-adaptation in mitochondrial metabolism [48]. In chickens, exposure to conspecific calls, and even music, *in ovo* stimulates neural and cognitive development resulting in enhanced spatial learning abilities in the days after hatching [49]. In utero learning of the maternal voice in humans is a manifestation of such a widespread biological phenomenon—it is presumably beneficial to the newborn infant to immediately orient to its mother as the likely primary caregiver immediately after birth.

Although the experimental evidence for such obvious functional adaptations in humans is relatively scarce, one compelling result is that there is a significant difference in the structural and functional development of the auditory system of pre-term infants compared to term infants. It is believed that this arises because of differential exposure to acoustic input, with pre-term infants being subjected to a substantially different auditory environment in a neonatal intensive care unit (NICU) compared to their term peers. It has been shown that exposure to recordings of the maternal voice and heartbeat during NICU stays leads to increased volume of primary auditory cortex at term compared to exposure only to standard hospital noise [50].

## Prenatal programming of human communicative behavior

A puzzling finding in the literature on human prenatal learning is that newborn infants produce cries that carry the pitch accent (or “melody”) of the language of their gestational environment. This accent is presumably learned primarily via exposure to the maternal voice. This intriguing observation was first published by Mampe and colleagues [51] who conducted a study comparing the cries of newborn full-term infants who gestated in either a monolingual French or monolingual German environment. They demonstrated that the spontaneous cries that they recorded from these infants 2–5 days *post-partum* had the melodic contour typical of speech in the environmental language, leading to the remarkable conclusion that the environmental language experienced in utero shapes the acoustics of infants’ cries. These findings have since been replicated in other linguistic environments. Wermke and colleagues [52] demonstrated that the fundamental frequency of cries was more variable in newborns whose ambient language was Lamnso, a complex tonal language of the Niger-Congo family, than in German newborns. In another study, Wermke and colleagues [53] compared the melodic contours of neonates who gestated in monolingual German and Mandarin Chinese environments, and found that the cries of Chinese newborns were more melodically complex than those of their German counterparts. This is consistent with the melodic contour of Mandarin (a tonal language) being more complex than that of German. Similarly, Prochnow and colleagues [54] analyzed the cries of Swedish newborns, and contrasted them with German newborns. Swedish is a pitch-accent language, and German is not. Swedish newborns produced cries of greater melodic complexity. Gustafson and colleagues have queried the methodology of some of this work [55], arguing that whilst individual infants’ cries are distinctive, the evidence for language-group differences is uncompelling. Nevertheless, a recent study using a neural network approach to classification showed that it was possible to accurately classify the native language of infants born in Germany or Japan to monolingual speakers of German and Japanese, respectively, based on samples of cries recorded at 2–6 days after birth [56]. Although this may leave some margin for *post-partum* learning, if these effects generalize, they constitute an extremely precocious expression of language-induced modification of cries.

Taken together, these studies indicate that a linguistic bias is present in vocal behavior extremely early in the infant’s *post-partum* life. Specifically, there is an impact of the melodic contour of the environmental speech on infants’ earliest communicative gestures (for the proposed underlying mechanisms, see Box 1). This is manifested in, but overlaid upon, the universal innate reflex of crying.

**Box 1. Substrates of audiomotor learning in the womb**

The neuroanatomical basis for an auditory-motor mapping is provided by the arcuate fasciculus, a white matter bundle that connects posterior temporal areas to inferior frontal areas, via the parietal lobule [57], which is thought to be instrumental in vocal learning, by providing the means for auditory feedback to tune articulation [58]. Newborns have a precursor of the arcuate fasciculus, similarly connecting auditory areas to premotor and somatosensory areas [59–61]. Although there is scant characterization of this connection prior to birth, from in utero, preterm or *ex vivo* samples, it must be present prior to term, laying the cerebral ground for a direct influence of auditory stimulation on the motor system, potentially for the entire last trimester of pregnancy, once the auditory system is functional. However, in the uterine environment the fetus cannot receive auditory feedback about vocalizations; generating an audible vocalization requires the creation of a pressure differential between air in the lungs and air outside the lungs, forcing air through the vocal folds. In utero, the fetus is immersed in a fluid-filled environment and consequently cannot engage in audible vocal behavior, and thus receives no auditory feedback on articulatory gestures that may be executed. Thus, auditory feedback is unlikely to contribute to in utero tuning of articulatory motor patterns. However, one source of feedback that is available is somatosensory feedback. If the fetus were to execute articulatory motor gestures, in utero somatosensory feedback would likely be received. Since pitch-contour and amplitude of infant cries are highly correlated [62], it is possible that if the fetus were to learn only to match its motor output with the typical intensity contour of the environmental speech input, this would provide a basis for developing articulatory motor control patterns that bias cry production towards the native accent. It is possible that the extraction of patterns from auditory stimulation depends upon an amodal representation of magnitude over time that is stored and can be retrieved in the service of multiple domains. Even if this is the case, the fetus has limited scope for movement that may recapitulate these patterns, due to the relative paucity of space in the womb. Indeed, other than the digits, only orofacial movements can readily be executed, again potentially biasing the system towards vocal motor learning.

The potential significance of such accented crying remains a matter of speculation. However, the discovery of accents in newborns’ cries has frequently been considered from the perspective of speech development, and indeed the melodic quality of infants’ cries has been shown to be predictive of language outcomes in the second year of life [63]. This tantalizingly suggests that whatever mechanisms enable precocious acquisition of a “native-like” vocal-motor repertoire are of substantial importance to language development, and, significantly, suggest that crucial determinants of speech acquisition in play during gestation, manifest in cry melody. However, another important question relating to this has not been addressed—does crying with a melodic contour that reflects the typical pitch-contour of the environmental (and likely native) language confer a benefit to the infant immediately, rather than to the future toddler? Does this precocious bias in vocal production serve as a signal to potential caregivers, identifying the infant as a member of their (linguistically-defined) community? Could the superposition of a native-like melody over a cry render that cry a superior signal for eliciting and optimizing caregiver responses, by maximizing salience and reducing aversiveness?

In addition to this theoretical possibility, recent ultrasound investigations during the third trimester have revealed that the fetus produces lip movements in response to maternal speech [64,65], and that when these lip movements occur, they are consistent with syllables enunciated by the mother. This is of particular interest as it suggests that the fetus may engage in an articulatory mirroring of auditory input. The likelihood that this is automatic, rather than voluntary, is suggested by the fact that it is generally thought the fetus is not conscious [66]—any suggestion of deliberate practice should be regarded cautiously. This provides a basis for elaborating a theory of how in utero experience of speech can drive the adaptation of the articulatory motor apparatus to have a bias towards producing natively-accented utterances. Namely, that the fetus possesses a mechanism that automatically maps speech sounds from the auditory to the motor domain, which contributes to developing articulatory-motor patterns executed at birth. Interestingly, similar data have been forwarded in non-human primates: a recent study [67] used ultrasound to demonstrate in utero practicing of vocal gestures that are to be produced after birth in marmosets. It should be noted that in both humans and non-human primates, whilst the work demonstrating these effects is limited in scale, and that further investigations are required to establish with certainty whether articulatory mimicry in utero is a reliable phenomenon, the presence of a vocal gestural repertoire (albeit a limited one) in utero provides an intriguing potential substrate for the influence of auditory input.

## Solving the mystery?

The arguments presented above do not solve the mystery of the dominance of the vocal mode. However, there is an overwhelming mass of evidence suggesting that prenatal auditory experience alters the developing brain and that it can do so in a manner that is consistent with programming for a phenotypic benefit for an individual. The existence of such a well-conserved mechanism across species (and across taxonomic groups, including amphibians, reptiles, birds, and mammals) suggests that the influence of the uterine environment must be considered in the story of human language evolution. The nature of expressive language is constrained by the available channels, and it is likely that the prenatal influences that drive toward the development of a dominant vocal mode also result in a reciprocal influence of the mode on the underlying form. If a co-evolution of production and perception is assumed, it is also probable that these co-evolved with the relevant cognitive architecture for encoding and decoding linguistic messages, and that the output modality will have exercised some constraints shaping the nature of the system itself. We hope that approaching this question using the novel imaging tools at our disposal and with a focus on enquiring into the potential mechanisms and results of prenatal learning will provide a framework for novel modes of enquiry into the key drivers implicated in the evolution of language.

Although the refrain “further work is needed” is worn past the point of cliché, it remains as true as ever in addressing the question “why do humans speak?”. We hope future work will shed further light on the proximal benefits incurred by crying. Whilst challenging, large-scale behavioral experiments investigating preferences (e.g., inviting ratings of aversiveness and salience) of cries showing the melody of listeners’ native languages can provide a first insight into whether adults are sensitive to this dimension. Further studies, for example investigating autonomic physiological responses, such as galvanic skin response or pupillometry, could also provide a means for covert evaluation of whether cry melody (or other features of the acoustics of cries) can have a substantive impact on adult listeners. Neuroimaging studies of adult responses to cries having different melodies could also shed some light on the neurophysiological consequences of exposure to cries conveying this potential signal of group membership. Additional comparative work, particularly in our closest living primate relatives, will also be instrumental in helping further unpack the evolutionary emergence of prenatal learning and the extent to which such abilities are unique to humans or rooted more deeply in the primate lineage. Finally, thanks to advances in non-invasive imaging technologies, in particular fetal fMRI, it is now possible to explore the cerebral bases of in utero perception and prenatal learning, which may bring us one step closer to understanding the impact of exposure to a rich auditory environment on the brain and its consequences for communicative behavior. Ultimately, it seems that elucidating the foundations of speech will require a concerted effort to understand what is happening even before birth.

## Abbreviations:

GA: gestational age
NICU: neonatal intensive care unit
